# Observations on the Conversion of Iron into Steel

**Published:** 1808-10

**Authors:** Joseph Priestley


### OBSERVATIONS *on the* CONVERSION *of* IRON *into* STEEL. *By the Rev.* JOSEPH PRIESTLEY, *LL. D.*

ACCORDING to the antiphlogistic theory, iron becomes steel by imbibing *carbone.* To this is said to be owing the addition to its *weight* in the process of cementation; and the black flakes which remain undissolved after the solution tion of steel in diluted vitrolic acid, is said to be *plumbago*, or carbone united to iron.

I cannot, however, help concluding, from some late experiments, that iron is converted into steel by imbibing only phlogiston from the charcoal with which it is cemented, and that the addition to its weight is not from *carbone*, but from *finery cinder*.

Having made a quantity of iron filings perfectly pure, by first expelling from them all the air that they would yield by heat, then washing out any carbonaceous matter that might be contained in them, and again exposing them to heat, I took 120 grains of them, and, heating them with a burning lens in inflammable air, they imbibed eighteen ounce-measures of it.

In consequence of this, from being of a dark colour, they became exceedingly bright, and I concluded that they had now become steel, though I could not prove it by any decisive experiment. But, after dissolving them in diluted vitriolic acid, a quantity of black matter, as from the solution of steel, remained unaffected by it. This being heated in inflammable air, imbibed a considerable quantity of it; and then, by means of diluted vitriolic acid, it gave inflammable air very copiously. This black matter, therefore, had evidently the properties of finery cinder.

I then dissolved 200 grains of broken *watch-springs*, which are undoubtedly pure steel, and collected from the solution three grains of black matter. Heating this inflammable air, it imbibed a great proportion of it, and then by means of the diluted acid, it gave inflammable air as copiously as iron or steel filings would have done. This black matter, therefore, was finery cinder, and not carbone or plumbago; and, as iron acquires weight by becoming finery cinder, and this weight I think I have proved to come from its absorption of *water*, it can hardly be doubted but that this addition of weight to iron, in becoming steel, is from the same cause. Indeed, I believe it to be impossible to expose iron to a red heat in circumstances in which there is any possible access of water, or of air which always contains water, without a partial calcination of it, that is, its becoming, superficially at least, finery cinder.

This was evidently the difference between the solution of the watch-springs, and that of an equal weight, viz. 200 grains) of broken *polished needles*, which had not undergone any calcination: for the black matter that remained mainded from the solution of them would not have weighed a quarter of a grain. Giving colour to steel (which is done to the watch-springs) is always a partial calcination of it, and this appears, from the preceding experiments, to be a conversion of part of the metal into finery cinder, which is the reverse of plumbago, being, according to the new theory, an *oxyd* in the highest possible degree; whereas, if plumbago contain any oxygen, it is in the lowest degree.

